# Obesity and the microbiome in atopic dermatitis: Therapeutic implications for PPAR-γ agonists

**DOI:** 10.3389/falgy.2023.1167800

**Published:** 2023-03-27

**Authors:** Jeremy P. McAleer

**Affiliations:** Department of Pharmaceutical Sciences, Marshall University School of Pharmacy, Huntington, WV, United States

**Keywords:** atopic dermatitis, microbiota, dysbiosis, obesity, PPAR-*γ*, probiotics, allergy

## Abstract

Atopic dermatitis (AD) is an inflammatory skin disease characterized by epidermal barrier disruption, Th2 immune responses to skin allergens and microbial dysbiosis within affected lesions. Studies within the past decade have revealed genetic and environmental factors contributing to AD in children. Obesity is a metabolic disorder that often manifests early in life and is associated with reduced bacterial diversity, leading to skin colonization with lipophilic bacteria and intestinal colonization with pro-inflammatory species. These changes impair epithelial barriers and promote Th17 responses, which may worsen the severity of AD symptoms. While few studies have examined the contribution of microbiota in obesity-induced allergies, there is emerging evidence that PPAR-γ may be an effective therapeutic target. This review discusses the microbiome in pediatric AD, treatment with probiotics, how disease is altered by obesity and potential therapeutic effects of PPAR-γ agonists. While healthy skin contains diverse species adapted for specific niches, lesional skin is highly colonized with *Staphylococcus aureus* which perpetuates the inflammatory reaction. Treatments for AD should help to restore microbial diversity in the skin and intestine, as well as epithelial barrier function. Pre-clinical models have shown that PPAR-γ agonists can suppress Th17 responses, IgE production and mast cell function, while improving the epidermal barrier and microbial homeostasis. Overall, PPAR-γ agonists may be effective in a subset of patients with AD, and future studies should distinguish their metabolic and anti-inflammatory effects in order to inform the best therapies.

## Introduction

Atopic march describes the successive development of allergic diseases beginning in infancy, including atopic dermatitis (AD), allergic rhinitis, asthma and food allergy (1). The first manifestation is usually AD, occurring in 85 percent of affected children before the age of 5 (2). In U.S. children, the prevalence of AD is 17 percent and is associated with a reduced quality of life due to anxiety and sleep disturbances. AD is typically caused by Th2 immune responses against skin allergens that lead to IgE production. Re-exposure to these allergens results in the degranulation of skin-resident mast cells that have been sensitized with IgE, leading to allergic manifestations including a rash, inflammation and pruritus. Longitudinal studies suggest that severe AD early in life increases the risk for allergic rhinitis or asthma in childhood or adulthood (2). These findings underscore the importance of identifying factors regulating the development of AD that may be exploited as therapeutic targets.

Epithelial surfaces of the body are colonized with microorganisms, collectively referred to as the microbiome. Many studies have characterized bacterial and fungal species present in the gastrointestinal (GI) tract, skin and lungs in healthy and diseased individuals, revealing several immunomodulatory functions. Nevertheless, our molecular understanding of how microbial colonization impacts the immune system is incomplete. Beneficial and detrimental roles for microbes have been identified in pediatric allergies, with protection associated with breastfeeding, vaginal delivery, having pets and avoiding antibiotics (3). Collectively, these studies suggest that dysbiosis, or imbalances in microbial species prevalence and diversity, contributes to atopy.

Obesity is a metabolic disorder that often manifests early in life and is associated with reduced bacterial diversity in the GI tract (4–6). Several lines of evidence suggest that obesity increases the severity of allergic diseases (7), including food allergies (8). While few studies have examined the contribution of microbiota in obesity-induced allergies, there is emerging evidence that PPAR-γ may be an effective therapeutic target. PPAR-γ is a lipid-sensing transcription factor that regulates genes involved in lipid metabolism, insulin sensitivity, adipogenesis and inflammation (9). Due to these functions, medications that stimulate PPAR-γ are approved for treating diabetes mellitus and inflammatory bowel diseases, underscoring its multi-functional roles. This review discusses the microbiome in pediatric AD, treatment with probiotics, how disease is altered by obesity and potential therapeutic effects of PPAR-γ agonists.

## Atopic dermatitis

### Pathophysiology

**Figure 1:**
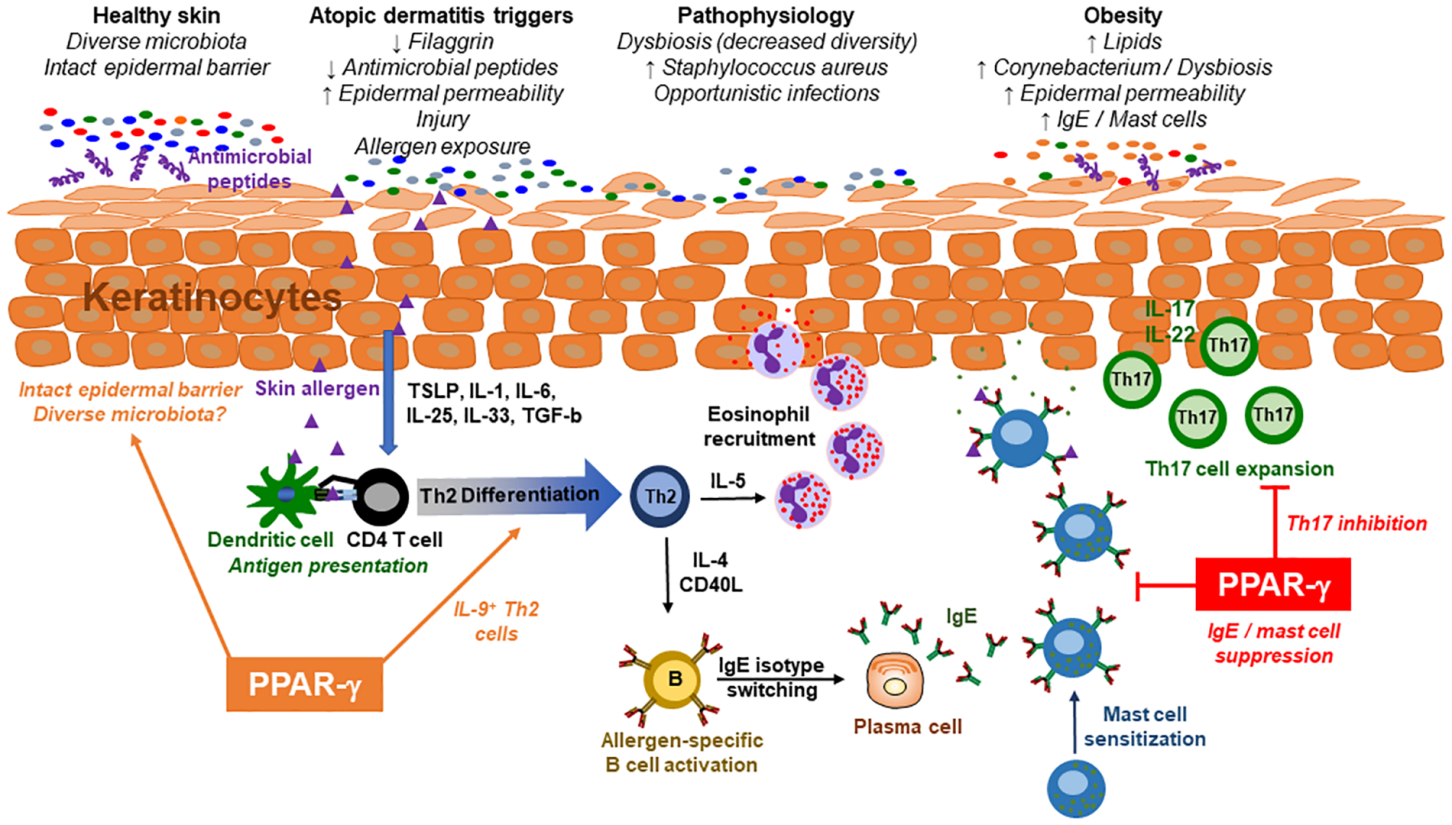
Interactions between the immune system and microbiome in atopic dermatitis. In healthy individuals, the epidermal barrier is intact and maintained by filaggrin expression, antimicrobial peptides, and other factors. This is associated with a diverse microbiome that colonizes distinct niches on the skin surface. Atopic dermatitis (AD) patients have an impaired skin barrier leading to increased permeability within the keratinocyte layers. This is associated with reduced microbial diversity, including increased colonization with Staphylococcus aureus. These individuals are at increased risk of inflammatory skin injury, leading to the production of cytokines that facilitate Th2 cell differentiation. Allergen-specific Th2 cells then produce cytokines that promote eosinophil recruitment to the skin and IgE production by B cells. Mast cells that are sensitized with IgE release histamine and other inflammatory mediators following subsequent exposures to the skin allergen, while eosinophils mediate skin damage by releasing intracellular granules. Obesity is associated with increased lipid composition on the skin which facilitates dysbiosis, including colonization with lipophilic Corynebacterium species. The chronic inflammatory milieu in obesity promotes Th17 responses on epithelial surfaces including the skin. Cytokines produced by Th17 cells, including IL-17 and IL-22, impact keratinocyte differentiation, epithelial permeability and antimicrobial peptide production. In addition, diet-induced obesity is associated with increased production of IgE and mast cell accumulation in the skin. These effects of obesity are thought to increase the severity of AD in affected patients. PPAR-γ is a transcription factor with anti-inflammatory properties that also regulates lipid metabolism. Medications that target PPAR-γ may treat AD through multiple mechanisms including suppression of Th17 differentiation, mast cell accumulation, IgE production or pro-inflammatory cytokines. PPAR-γ agonists also improve insulin sensitivity, lipid metabolism, epidermal barrier function and microbial diversity, while pro-inflammatory effects include Th2 differentiation and IL-9 production. It is important to ascertain if the efficacy of PPAR-γ agonists in microbiota-dependent allergic diseases is influenced by body mass index, comorbidities or potential pro-inflammatory effects on the immune system in certain patient endotypes.

Atopic dermatitis (AD), or eczema, is a chronic, relapsing inflammatory skin disease with a prevalence of up to 25% in children and 7% in adults (10). Symptoms beginning in childhood may subside in adolescence or continue for years, involving periods of exacerbation and remission. Affected individuals have dry, cracked skin, intense pruritus and a erythematous rash due to Th2 immune responses against allergens (Figure 1). The skin barrier is impaired within crusted erythematous areas, associated with epidermal hyperplasia, scaling and lichenification (10). Some individuals have deficiencies in filaggrin or antimicrobial peptides, increasing permeability of the skin and susceptibility to opportunistic infections, respectively. Skin injury often precipitates AD, causing keratinocytes to produce cytokines that promote inflammation and immune activation (TSLP, IL-1, IL-6, IL-25, IL-33, TGF-b) (10). Th2 cells play a central role in driving pathogenesis, leading to allergen-specific IgE production and eosinophilia within affected lesions (Figure 1). Th17 cells also have a role, as lesional regions have increased expression of inflammatory genes including *IL13, IL17A, IL17F, IL22, CCL17* and *S100*s (11). During infancy, atopic sensitization is also associated with *IL9*, *IL33* and *IL33R* expression. Scratching affected regions leads to further impairment of the epidermal barrier, increasing susceptibility to opportunistic pathogens including *Staphylococcus aureus*.

Obese patients with immunologic diseases including atopy and asthma have more severe disease than their lean counterparts (7, 12). Studies examining obesity in infancy and childhood also found a positive association with the prevalence of AD (13, 14). Gender differences have been identified, as only females with AD had higher abdominal obesity rates than healthy controls (15). Leptin deficiency is commonly associated with obesity; however, conflicting studies suggest it may not be directly involved in AD pathogenesis (16). A murine model investigating the mechanism of obesity-driven AD identified a role for Th17 cells (17). Although neutralization of Th2 cytokines (IL-4, IL-13) protected lean mice from AD, this treatment exacerbated disease in obese mice due to the lack of PPAR-γ expression in CD4 T cells (17). In addition, the PPAR-γ agonist rosiglitazone reduced AD severity in obese mice, demonstrating a protective anti-inflammatory function (17). While few studies have examined the role of microbiota in obesity-induced AD, it may contribute to the persistent low grade systemic inflammation that occurs in obese children (18).

### Skin Microbiota

**Figure 2:**
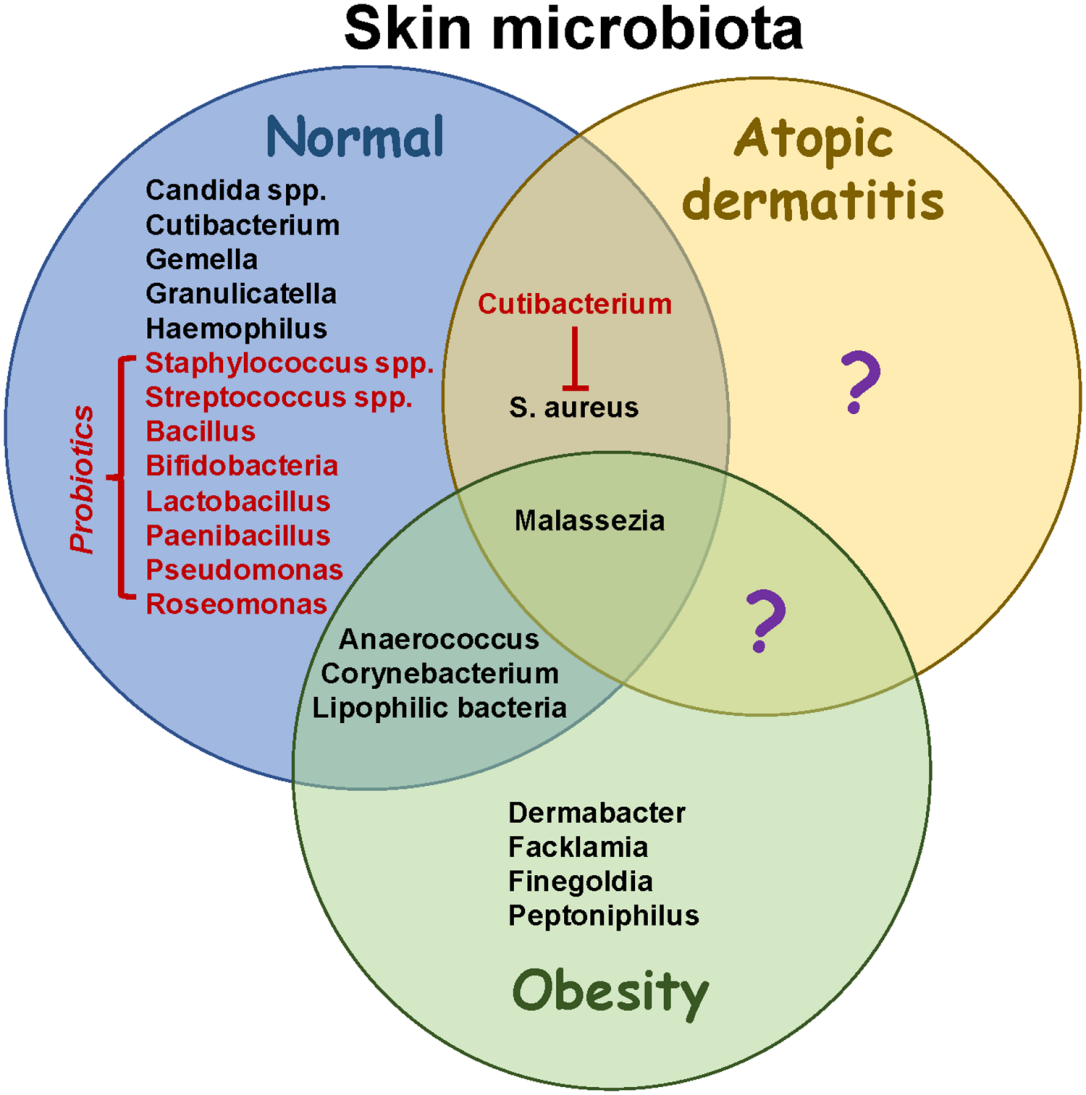
Microbiome changes in obesity and atopic dermatitis. Skin microbiota in healthy individuals has been well characterized, although more studies are necessary to elucidate developmental changes occurring throughout life. Structural changes associated with atopic dermatitis reduce bacterial diversity and increase colonization with Staphylococcus aureus. Potential probiotics (red font) help to restore diversity and have anti-inflammatory effects. In addition, they may suppress the growth of S. aureus. Obesity results in increased colonization with lipophilic species including *Corynebacterium*. While intestinal microbiota have been shown to contribute to AD severity in obesity (not shown), the role of skin microbiota in pathophysiology needs to be further studied. Identifying skin species that are unique to atopic dermatitis in the presence or absence of obesity (question marks) may help to guide therapeutic strategies to promote healthy microenvironments colonized with symbiotic microbiota.

Healthy skin microbiota contains diverse species adapted for specific niches, including *Cutibacterium, Malassezia, Staphylococcus* and *Corynebacterium* (Figure 2) (19). The most prevalent bacteria in healthy skin include *Cutibacterium acnes, Staphylococcus epidermidis*, and *Streptococcus mitis/oralis/pneumoniae/sanguinis* (20). In addition, young children have increased colonization with *Streptococcus, Granulicatella, Gemella, Rothia, Haemophilius* and *Candida spp.*, whereas *Cutibacterium, Corynebacterium, Staphylococcus, Lactobacillus, Finegoldia* and *Anaerococcus* are more abundant in adults. Over 100 fungal species have been identified on healthy skin, with most belonging to the phyla *Ascomycota* (21). Increased sebum production and structural changes after puberty may facilitate colonization with lipophilic microbes including *Cutibacterium, Corynebacterium* and *Malassezia*, replacing *Streptococcus* and *Candida* (20, 22). Thus, the skin microbiome in teenagers is more similar to adults than children. The overall diversity of skin microbes in children is due to specific environmental niches promoting colonization with certain species, which may change after the onset of puberty.

Few studies have analyzed the impact of obesity on skin microbiota. Having a low body mass index (BMI) correlated with an increased Shannon Diversity Index compared to normal weight or obese individuals (23). Ten genera were enriched in underweight people, including Gordina, Lupinus and Prevotella, whereas seven were enriched in obesity including Anaerococcus, Finegoldia and Peptoniphilus (Figure 2). In addition, Corynebacterium colonization correlated with BMI (23). A mouse study found that skin Corynebacterium species and free fatty acids increased in response to a high fat diet (24). The authors speculated that increased adipogenesis created a microenvironment that favored colonization with lipophilic bacteria such as Corynebacterium. Dietary factors have also been shown to influence skin bacteria in humans (25). These data suggest that BMI and diet impact the composition of skin microbiota. Further studies are needed to analyze skin fungi in obesity, as *Malassezia spp.* are lipophilic and can induce Th17 responses (26), which may contribute to obesity-driven AD (17).

AD is characterized by reduced lipid content in skin, higher pH and increased transepidermal water loss, which may shape the composition of microbiota. Although children with AD had a more diverse microbiome in non-lesional skin compared to adults, dysbiosis occurred within skin lesions due to an impaired barrier (20). Dysbiosis was associated with reductions in *Streptococcus*, *Cutibacterium* and *Malassezia*, accompanied by increases in *Staphylococcus aureus*, suggesting an antagonistic relationship between skin commensals (20, 21). *Streptococcus* may inhibit *S. aureus* growth by producing hydrogen peroxide (27), whereas *Cutibacterium* and *Corynebacterium* are involved in porphyrin metabolism which may further suppress *S. aureus* colonization (20, 28). Dysbiosis might also be caused by a failure in antimicrobial peptide production which leads to increased colonization with *S. aureus* (29), immune activation in response to superantigens (30), and the development of AD (31). Patients are also sensitized to fungal antigens, including those from *Malassezia spp.*, due to the disrupted epidermal barrier (21). Several lines of evidence implicate *S. aureus* in perpetuating AD. For instance, skin colonization at 3–6 months of age increases the risk of developing AD (32, 33), and both *S. aureus* and *S. epidermidis* increase during flares and decrease post-flare (34). Treating *S. aureus*-infected lesions with antibiotics reduces inflammation, demonstrating a critical role for microbiota in driving AD (35, 36). Topical treatments including corticosteroids, antibiotics and calcineurin inhibitors were associated with an increased diversity, including colonization with *Streptococcus, Cutibacterium* and *Corynebacterium spp* (34). Therefore, local inflammation during AD flares disrupts the microbiome by generating an environment that favors colonization with *Staphylococcus spp*. An adult study found that AD severity positively correlated with *S. capitis* and *S. lugdunesis* in lesional skin, and negatively correlated with *S. hominis* (37). Collectively, these observations suggest that factors influencing microbial colonization may impact an individual's susceptibility to AD.

Filaggrin (FLG)-deficiency contributes to dysbiosis by increasing skin pH, facilitating colonization with *S. aureus* (38). Virulence factors produced by *S. aureus* may then cause further breakdown of the skin barrier and stimulate immunity towards skin allergens (19). Skin microbiota are critical for the inflammation associated with FLG-deficiency. For instance, Flg^−/−^ mice have spontaneous dermatitis and increased colonization with *Staphylococcus spp.* (39). In this model, dermatitis was dependent on IL-1β, but independent of Th2 cytokines. When raised germ-free, Flg^−/−^ mice showed signs of dermatitis as neonates; however, this inflammation resolved in adulthood (39), suggesting that dysbiosis maintains the chronic inflammation in genetically susceptible individuals. Other genes associated with childhood AD include *GRP1*, *CCL22*, *TTC27* (40), although their impact on skin colonization remains unclear.

Studies have explored if topically-applied probiotics can alleviate inflammation by restoring homeostasis. Ito, et al. demonstrated that skin inoculation with *S. cohnii* protected against spontaneous and chemical-induced AD by suppressing inflammation (41). Protection was attributed to the expression of glucocorticoid-inducible genes in skin, although *S. cohnii* strain-specific differences were observed. *Roseomonas* and *Cutibacterium spp.* may inhibit colonization with *S. aureus* and be suitable probiotics for AD skin (19); however, some *Cutibacterium spp.* (*C. acnes*) also facilitate *S. aureus* biofilm formation. A probiotic formulation containing *Roseomonas mucosa*, poly(vinyl pyrrolidione), poly(vinyl alcohol) and sodium alginate demonstrated antimicrobial activity against *S. aureus* (42). Other topical probiotics investigated for skin use include *Bacillus*, *Bifidobacteria*, *Lactobacillus, Paenibacillus, Pseudomonas, Staphylococcus, Streptococcus*, and others (43). These studies demonstrate a focus towards treating dysbiosis in order to reduce skin inflammation.

### Gut microbiota in atopic dermatitis

The gut microbiome has been compared between infants with and without AD. Facultative anaerobes predominate during the first 6–12 months of life prior to colonization with obligate anaerobes (44). Notably, the anaerobe *Akkermansia muciniphila* was only detected in healthy infants and their mothers, suggesting it may correlate with protection against AD (45). Breast feeding may account for some of the microbiota differences between AD and non-AD infants (44). In the second year of life, moderate to severe AD was associated with a higher abundance of facultative anaerobes compared to healthy controls. This correlated with decreased production of the short chain fatty acid butyrate, and decreased expression of the butyrate receptor *Gpr109a* and *Pparg* in the colon of AD-induced mice (44). The authors speculated that low butyrate levels perturb the microbiome by decreasing oxygen consumption, promoting the growth of facultative anaerobes. In support, metabolic pathways responsive to oxidative stress are upregulated in the microbiome of AD patients (46). This was associated with increased colonization with *Faecalibacterium prausnitzii* and decreased levels of butyrate and propionate. In addition to *Faecalibacterium*, *Bacteroides* and *Ruminococcus lactaris* are increased in AD infants, whereas *Bifidobacterium*, *Clostridium paraputrificum* and *Lachnospiraceae* are decreased (45). Taken together, these data demonstrate that AD is associated with an altered microbial profile in the GI tract.

Several bacterial species are being tested for their therapeutic efficacy in AD. Oral administration of *A. muciniphila* or *F. prausnitzii* improved AD symptoms in mice, including dermatitis score, scratching behavior, serum IgE and TSLP (47). Treatment with these strains increased filaggrin in skin and ZO-1 expression in the intestine, demonstrating improved epithelial barriers. The mechanism of how *A. muciniphila* protects against AD may be multi-factorial, as monocolonization of germ-free mice upregulated genes involved in epithelial homeostasis, antigen presentation, immune activation and PPAR-α-dependent metabolism (48). Oral treatment with *Pediococcus acidilactici* decreased AD severity in mice, including erythema, hemorrhage, edema, excoriation, dryness and scratching behavior (49). In addition, *P. acidilactici* prevented the AD-induced decreases in *Lactobacillales, Butyricicoccus*, and *Ruminococcus*, demonstrating that it may help to restore intestinal homeostasis. Oral administration of *Lactobacillus paracasei* reduced AD-associated skin lesions, epidermal thickening, serum IgE and immune cell infiltration into skin lesions in a mouse model of AD (50). This was associated with decreased effector T cell cytokines and increased IL-10 and TGF-β. Similar results were found for *L. plantarum*, including increased colonization with butyrate-producing bacteria (51). In children with mild or moderate disease, *L. plantarum* supplementation resulted in a greater reduction in AD scores compared to placebo (52). A mouse model using *Limosilactobacillus reuteri* found that combining prenatal and postnatal treatment was better at improving AD and lowering serum IgE than postnatal treatment alone (53). This suggests maternal factors may influence the risk of developing AD *in utero*. Supplementation with *L. reuteri* increased microbial diversity in the GI tract, including colonization with *Faecalibacterium, Bifidobacterium* and *Akkermansia* (53). The anti-inflammatory effects of *L. reuteri* may have been due to PPAR-α signaling and retinol metabolism. These data demonstrate complex roles for microbiota and probiotics in protection against AD, including suppressing inflammation, improving microbial diversity, and increasing epithelial barrier function.

### Gut microbiota and obesity

Few studies have examined the role of gut microbiota in obesity-induced allergies. Altering the microbiome with antibiotics during the first year leads to increased adiposity (54), demonstrating a role for bacteria in regulating metabolism. In support, human microbiota from obese donors increase body weight and adiposity when transferred to germ-free mice (55). Currently, a clinical trial is analyzing the microbial signature associated with obesity and AD (56). A mouse study demonstrated that diet-induced obesity aggravates contact hypersensitivity in an IL-17-dependent manner (57). This correlated with colonization of the GI tract by pro-inflammatory species including segmented filamentous bacteria, *Clostridium type IV*, and *Enterococcus*. Another study found that high fat diets increase IgE, small intestinal mast cells and gut permeability in response to food allergens (58). These data demonstrate that obesity-induced changes to the microbiome correlate with AD severity, and suggest that probiotics associated with leanness may protect against AD. Several species were shown to have lipid-lowering effects in human epidermal keratinocytes *in vitro*, including *Bifidobacterium bifidum, Lactobacillus acidophilus, L. delbrueckii, L. casei*, and *L. gasseri* (59), although their impact on obesity and atopy remain unknown. In vivo, *Bifidobacterium breve* persisted for at least 90 days after administration, whereas *Lactobacillus salivarius* colonization was transient (60), demonstrating that some probiotics may need more frequent administration than others.

### PPAR-γ and childhood obesity

PPAR-γ is a master regulator of adipogenesis and functions as a transcription factor, improving insulin sensitivity (61). In addition, PPAR-γ has anti-inflammatory functions through its suppression of NF-κB and cyclooxygenase 2 in epithelial cells, granulocytes and T cells. High expression levels within adipose tissue, intestinal and immune cells contributes to the therapeutic efficacy of PPAR-γ agonists in type 2 diabetes and inflammatory bowel diseases (IBDs). Natural ligands for PPAR-γ include prostaglandins, medium to long chain fatty acids, foods and environmental pollutants (62). Genome studies have identified *PPARG* as one of the genes linked to childhood obesity (63, 64). While a dominant negative mutation is associated with severe insulin resistance, type 2 diabetes and hypertension (65), a gain of function is linked to extreme obesity (66). In mice, the dominant negative mutation exacerbates insulin resistance in the context of leptin-deficiency, demonstrating antagonistic roles for leptin and PPAR-γ in adipogenesis (67). PPAR-γ concentrations in obese children positively correlate with birth weight, but negatively correlate with waist circumference (68). Further, *PPARG* expression in immune cells and adipose tissue negatively correlate with obesity (69, 70), demonstrating complex functions for this transcription factor in regulating body weight. TMEM18 induces the expression of *PPARG* in adipose tissue and is critical for adipocyte differentiation (69). The inflammatory cytokine TNF suppresses both *TMEM18* and *PPARG1*, leading to increased adipocyte size, decreased adiponectin, decreased insulin sensitivity and macrophage infiltration into adipose tissue. Some of the effects of PPAR-γ activity are mediated by adiponectin and ANGPTL4 (71, 72), with tissue-specific functions identified in the GI tract, skin, adipose tissue and immune cells (17, 73–79). Notably, microbiota have been found to regulate PPAR-γ signaling in the gut (75), although the role of microbiota on extra-intestinal functions of PPAR-γ are less clear.

### PPAR-γ as a therapeutic target in atopic dermatitis

Due to its expression profile and anti-inflammatory effects, PPAR-γ may be a potential therapeutic target in microbiota-dependent diseases. The thiazolidinedione (TZD) rosiglitazone alleviated AD in response to a high fat diet, demonstrating a critical function for PPAR-γ (17). In this model, diet-induced obesity increased the severity of AD in an IL-17-depedendent manner. This was attributed to dietary fat downregulating *Pparg* within Th2 cells, allowing for the expansion of Th17 cells (17). A model of atopic march found the combination of rosiglitazone and dexamethasone suppressed allergic skin inflammation better than either medication alone, suggesting TZDs may synergize with glucocorticoids (76). Topical treatment also reduced subsequent lung inflammation following intranasal challenge. While it remains to be determined if PPAR-γ alters the microbiome in AD, *Pparg* expression is decreased in the colon of mice with AD, correlating with dysbiosis (44). Further, PPAR-γ agonists modulate intestinal microbes associated with Western diets or colitis (80–83). The mechanism through which PPAR-γ regulates GI microbiota involves suppressing lactate fermentation and promoting beta oxidation (84), facilitating colonization with anaerobes. Although PPAR-γ stimulation affects microbiota, therapeutic effects of agonists most likely arise from the suppression of Th17 cells (17), epidermal keratinocyte growth (85), mast cell development and differentiation (86, 87), and IgE production (88), demonstrating several anti-inflammatory functions that may benefit AD patients (Figure 1).

The etiology and pathophysiology of psoriasis bears some similarities with AD, including obesity, microbial dysbiosis and Th17-mediated inflammation (89). Clinical studies identified anti-psoriatic effects of TZDs (90), suggesting these medications may help in AD. Nevertheless, the skin microbiome is distinct in psoriasis and characterized by *Corynebacteria spp.* rather than *S. aureus* (91). Further, only AD patients have transcriptome signatures associated with epithelial barrier function and immune activation, suggesting that TZDs must be further studied before their use in pediatric AD patients. TZDs have a black box warning for congestive heart failure, a rare but serious side effect (92). Other adverse reactions include edema, weight gain, anemia and bone fractures. The use of mesalamine for IBD has been associated with renal impairment, hypersensitivity and photosensitivity. Further, PPAR-γ can exert pro-inflammatory effects through IL-9 which exacerbates dermatitis (93, 94). Although few studies have assessed the clinical use of PPAR-γ agonists in children, no serious adverse effects were reported in trials for IBD and autism (95, 96), suggesting an acceptable safety profile. Collectively, pre-clinical studies suggest that PPAR-γ agonists might be effective in a subset of patients with AD, and trials comparing their local vs. systemic administration may help to minimize the incidence of adverse reactions.

## Conclusions

A surge of studies from the last decade helped to define the commensal microbiome in pediatric AD and how dysbiosis contributes to chronic diseases. Recent findings into the pathophysiology of obesity-driven AD have revealed PPAR-γ to be a potential therapeutic target. Future studies examining the efficacy of PPAR-γ modulators should distinguish their metabolic vs. anti-inflammatory effects in the gut, skin, adipose tissue and immune system, in order to inform the best therapies. The severity of AD and its role in initiating atopic march underscore the significance of utilizing effective treatments which may help to reduce the risk of asthma later in life.
